# Supplementary material for: Are carbon emissions trading and green financial instruments synergistic? -Comprehensive quantitative research based on content analysis
Source: PLoS One. 2024 Mar 7;19(3):e0298601. doi: 10.1371/journal.pone.0298601 (PMC10919601; doi:10.1371/journal.pone.0298601)
Supplement: S1 Data — (ZIP) [file pone.0298601.s001.zip › Data/地级市Z_F工作报告.zip!/╜¡╦╒/┴1⁄4╘╞╕█╩╨/└┤╫╘╣1⁄2╓┌║┼í╛┬φ┐╦╩2╛▌═°í┐_2012.docx]

各位代表：

现在，我代表市人民政府向大会作工作报告，请予审议，并请各位政协委员提出意见。

2011年工作情况

2011年是十二五开局之年，全市人民在中共连云港市委的领导下，紧扣主题主线，深入实施沿海开发，统筹推进各项工作，经济社会发展取得新成效。预计实现地区生产总值1410亿元，增长13%左右。完成财政总收入462亿元、一般预算收入180亿元，分别增长31.2%和27.4%。全社会固定资产投资、社会消费品零售总额、出口额分别增长24.7%、17.3%、42.3%。城市居民人均可支配收入、农民人均纯收入分别增长14%和16%。完成了市十二届人大四次会议确定的年度目标任务。

一年来的工作及成效主要有以下几个方面：

一、沿海开发深入推进

以港口为龙头的基础设施加快建设。按照沿海开发三年行动方案，大力推进港口航道、码头和集疏运体系建设。30万吨级航道完成投资14.2亿元，连云港区具备20万吨级船舶通航条件，徐圩港区5万吨级航道主体完工。连云港区氧化铝、散化肥泊位具备投产条件，徐圩港区30万吨级原油码头项目完成工可报告，赣榆港区4个5万吨级码头开工，燕尾港3万吨级码头竣工。集疏运体系日益完善，铁路电力机车直通进港，东疏港道路建成通车。全面推进交通、水利、电力等基础设施建设，204国道、242省道、临海高等级公路建设进展顺利，蔷薇河下游清淤、大型泵站改造加快实施，500千伏伊芦、220千伏灌河输变电工程建成使用，沿海开发的硬件支撑能力不断增强。

开发园区产业承载功能有效提升。引导开发园区专业化、特色化发展，市开发区五园五中心建设有序推进，国家新能源产业和中小企业科技创新与成果转化示范园加快建设，恒瑞研发中心建成使用，康缘、豪森等一批医药产业园完成主体工程，中日纳米研究院揭牌成立。徐圩新区基本形成四纵四横路网框架，珠江钢管、宝通镍业等一批临港产业项目加快实施，产业服务中心等功能平台建设取得积极进展。东海硅材料、赣榆海洋产业、灌南船舶、灌云农业机械等特色园区转型升级步伐加快，省级共建园区增至7家，省级以上开发区工业增加值增幅高于全市16.2个百分点。

重大事项取得进展。经过努力，东中西区域合作示范区获得国务院批准设立，成为首个国家级区域合作示范区。积极参与省沿海产业投资基金组建工作，编制完成省级金融改革创新综合试验区规划。徐圩新区规划环评通过审核。中日（连云港）生态科技产业园建设、中德中小企业园申报取得进展。国家交通电子口岸分中心挂牌运营，全国焦炭期货交割库获批设立。两翼口岸开放列入国家发展规划。大力争取炼化一体化、液化天然气项目列入国家十二五专项规划。我市成为全国主体功能区规划重点开发区域，政策优势进一步凸显。

二、产业经济持续较快增长

工业主体地位不断增强。加快转型升级步伐，积极推动基础产业做大、新兴产业做优、支柱产业做强。完成工业投资750亿元，工业增加值增长17%，对经济增长贡献率提高1.8个百分点。组建江苏沿海钢铁集团，硅材料、盐化工等产业链条不断延伸，新医药、新材料、新能源等新兴产业快速发展，临港产业和新兴产业产值均突破1000亿元。着力增强骨干企业带动能力，实施百企培育工程，规模企业达到1228家，过10亿元、100亿元企业分别达到48家和3家。加大扶持力度，设立科技型中小企业信贷风险补偿专项资金，新增中小企业贷款88亿元，占全部新增贷款比重达到60.4%。坚持把节能减排作为促进转型升级的抓手，推进传统产业升级改造，实施重点节能改造项目40项、重点污染减排项目37项，申报省级资源综合利用产品28个，港口船用岸电系统成为全国水运节能减排推广项目。

现代服务业加快成长。港口新开加密集装箱航线14条，新辟霍尔果斯、喀什过境通道2条，新增内陆物流场站1家，铁水联运成为国家首批示范项目，完成吞吐量1.66亿吨、集装箱运量485万标箱，港口服务中西部的能力进一步提升。民航新辟航线2条，旅客吞吐量突破50万人。大力拓展旅游市场，游客接待量、旅游总收入分别增长17%和23%。加快商贸设施建设，沃尔玛、家乐福超市和义乌小商品城、华东物料城一期等建成开业。启动市区农贸市场改造，新改建平价商店45家、农家店209家。浦发银行连云港分行等10家金融机构开业，实现金融生态县全覆盖。信息服务、文化创意等产业取得新成效。

高效农业快速发展。克服冬春连旱不利影响，实现粮食生产九连增。实施农业现代化工程，加强农田水利建设，推进农业综合开发，新增高效农业31万亩、高效渔业10万亩，畜禽规模化养殖比重提高9个百分点。扶持特色农业发展，设施蔬菜、优质水果、苗木花卉、泥鳅养殖等规模不断扩大，东海县获得中国花木之乡称号，灌南县工厂化食用菌产业集聚度全国领先。推进农业产业化进程，新增省级农业产业园区4家、龙头企业5家。强化农产品质量安全监管，新增省级名牌农产品4个、无公害农产品基地45万亩。完成农产品出口3.4亿美元，增长14.5%。完善基层农业服务体系，实现农机专业合作社乡镇全覆盖。

三、城乡统筹加快推进

城市形象稳步提升。协调推进新区开发和旧城改造，中心城市建成区面积扩大到130平方公里。城市化率达到53%。东部城区展现新的海滨风貌，连云新城新增陆域面积10平方公里。新海城区加速互联东进，科教创业园区等新区开发稳步推进。实施南极北路、陇海东路等旧城改造项目57个，完成拆迁改造面积60万平方米。昌圩湖、玉带河等景观工程基本完成，新增城市绿地520万平方米。进一步加强城市管理，数字城管开通运行。四城同创扎实推进，园林城市创建通过国家考核验收。

城市功能不断完善。推进城市重点工程建设，金海大厦、金港湾大厦竣工使用，润科现代服务业中心基本建成。强化城市基础设施建设，海湾路、汇海路大桥等一批道路桥梁工程完工，自来水深度处理工程、南城污水处理厂建成试运行，新建改建自来水管道40公里、污水管网20公里，污水处理率82%。完善与居民生活密切相关的配套设施，修建便民道路30条，整治人行道板2万平方米，新增人防工程16万平方米，新开优化公交线路16条，城市对人口、产业的承载服务能力进一步增强。

城乡环境持续优化。强化城乡统筹规划的引领作用，推动城乡一体化发展。四县县城彰显个性魅力，加快向中等城市迈进。新型城镇化水平不断提升，一批具有特色规模的小城镇加快形成，新增省级康居示范村4个、新农村建设先进村15个。积极兴办农村实事，建成农村公路884公里，城乡客运一体化率85%，行政村客运班车通车率99.5%。解决了37万农村人口饮水安全问题。加强环境整治和生态保护，完成119个村庄环境综合整治和258条县乡河道疏浚，完成绿化造林13.7万亩，环境质量综合指数83.4。海州湾国家级海洋公园获批，海洋保护区面积达到681平方公里。

四、创新发展能力明显增强

重点领域和关键环节改革不断深化。全面实施事业单位岗位设置管理和绩效工资制度。实现政府办基层医疗卫生机构基本药物制度全覆盖。加快推进投融资体制改革，12家政府投融资平台转化为特殊类贷款公司，新组建市保障性住房投资公司，重组交通建设、城市建设和润财投资公司，对公共项目的支撑能力得到提升。加大直接融资力度，发行短期融资券15亿元，3家企业进入证监会发审程序。深化农村综合改革，新增土地流转面积57万亩，新增农民合作组织483家，农村经济发展活力明显增强。

开放水平进一步提升。努力克服各种不利影响，加大招商引资力度，积极开展城市宣传和项目推介，引进外资6.6亿美元，内联客方到位资金525亿元。落实出口退税政策，支持企业开拓国际市场，外贸进出口总额突破70亿美元。鼓励企业开展境外投资，加强外派劳务市场管理和应急处置。推进区域交流合作，融入长三角一体化发展，深化南北共建机制，做好对口援疆工作。海关、海事、国检、边检等单位加强整体联动，口岸服务进一步优化。外事、侨务、台湾事务等部门积极发挥促进开放作用，与韩国群山市、光阳市缔结友好城市。

创新要素加快汇聚。深入实施创新驱动战略，努力在更高起点上推动跨越发展，获批成为国家创新型试点城市，蝉联全国科技进步先进市。加大创新政策支持力度，财政科技投入增长45%。加强重大研发平台建设，省海洋资源开发研究院一期工程通过验收，建成东海硅材料等国家和省质量检测中心3个，新增科技公共服务平台18个。高校创新载体与地方产业融合发展，实施产学研合作项目300个。企业自主创新能力不断提升，70%以上大中型企业设立了研发机构，国家新药创制科技重大专项获批项目位居全国同类城市前列，恒瑞伊立替康成为国内首个通过美国食品药物管理局认证的注射液，康缘荣获中国驰名商标。高新技术产业产值增长45%。实施人才集聚工程，引进硕士以上人才749名，27名创新创业领军人才和79名高层次人才获得专项资助，落实人才安置房100套。

五、民生和社会事业协调发展

民生工作扎实推进。大力发展民营经济，以创业就业带动富民，实施全民创业助推行动、就业再就业援助行动，新增农村劳动力转移就业4.8万人，城镇新增就业7.5万人。千方百计增加城乡居民收入，全市最低工资标准提高17.7%，企业退休人员养老金人均月增加131元，城乡低保人均月提高到300元和210元。持续增强社会保障能力，公共财力用于民生领域的比重达66.7%。城镇职工养老、医疗、失业、工伤、生育保险参保人数平均新增1.6万人以上，覆盖率达95%以上。新农保、新农合参保率均达99.5%。大力实施安居工程，建成各类保障性住房9899套，发放廉租住房租赁补贴2917户。实施脱贫攻坚工程，提前一年基本消除农村绝对贫困现象。完善社会救助网络，提高城乡居民医疗救助比例，健全价格动态补贴机制，开展慈善助困，特殊困难群众基本生活得到保障。

社会事业协调发展。积极推进教育现代化创建工作，小学初中入学率均达100%，学前三年入园率、初中毕业生升学率稳定在96%以上。中小学校舍安全工程有序推进，连云港高级中学建成使用，创建省四星级普通高中2所。连云港中医药高职校完成迁建，江苏师范大学连云港校区挂牌招生。加强基本医疗卫生体系建设，新增三级综合医院2家，组建市级医疗集团3个，卫生监督机构全部达到省标。深入实施文化惠民工程，市博物馆、图书馆、美术馆、文化馆免费开放。全民健身活动广泛开展，在第七届城运会上取得优异成绩。全面做好人口管理服务，妇女儿童、老龄、关心下一代、残疾人及民族宗教工作得到重视和加强，档案、地方志、气象、防震减灾、老区开发事业不断发展。

精神文明建设和社会管理创新成效显著。大力培育社会文明新风尚，加强未成年人思想道德建设，广泛开展社会志愿者行动，市革命纪念馆建成开馆，开山岛夫妻哨入选2011年感动中国候选名单。加强基层民主政治建设，村委会换届选举工作顺利完成，县乡换届工作扎实推进。加强社会管理创新，不断深化平安连云港、法治连云港创建活动，积极预防和化解社会矛盾纠纷，完善信访维稳协调处置机制，社会大局保持和谐稳定，公众安全感达到90%以上。加强食品药品综合监管，开展食品非法添加、地沟油等专项整治，顺利通过省食品安全考核评估。落实安全生产责任制，强化校车等重点领域安全监管，安全生产形势保持稳定。实施基层应急管理五个一示范工程，突发事件防范应对能力得到加强。国防动员、民兵预备役和优抚安置工作稳步推进，实现全国双拥模范城六连冠。

一年来，我们认真履行人民赋予的权力，自觉接受市人大及其常委会的法律监督、工作监督和市政协的民主监督，认真听取人大代表、政协委员意见和建议，办结人大代表建议143件、政协委员提案427件。切实加强政府自身建设，大力推进政府职能转变，不断完善议事决策机制，努力提升决策水平。全面推进政务公开，加强行政权力网上公开透明运行，改革公文办理制度，提高行政效能。坚持勤政廉政，强化行政监察和审计监督，加大督查考核力度，促进工作落实。

各位代表，回顾过去一年的工作，全市上下抢抓机遇、凝心聚力、真抓实干、锐意进取，实现了十二五良好开局，成绩来之不易！这是中共连云港市委正确领导、全市人民团结奋斗、社会各界共同努力的结果。在此，我代表市人民政府，向全市广大干部群众，向各位人大代表、政协委员和离退休老同志，向各民主党派、工商联、人民团体和无党派人士，向驻连部队、武警官兵、公安干警和驻连部省属单位，向热心支持连云港建设的海内外各界人士，表示崇高的敬意和衷心的感谢！

在总结成绩的同时，我们也清醒地看到，全市发展仍面临不少困难和问题，政府工作还存在一些不足和差距。主要表现为：经济总量依然偏小，结构性矛盾较为突出；资源环境约束加剧，节能减排压力加大；外部经济环境趋紧，利用外资难度增加；城市功能还不完善，规划建设管理方面还有不少薄弱环节；城乡居民持续增收压力加大，改善群众生活还有大量工作要做；投资发展环境仍需改善，创业的氛围不够浓厚，少数部门和单位服务发展的主动性、执行制度的严肃性还不够强，政府整体执行力还需进一步提高。对于这些问题，我们将高度重视、认真解决。

2012年工作意见

2012年是党的十八大召开之年，是全面落实省市党代会战略部署、实现沿海开发三年突破的决战之年，也是推进全面小康社会建设、加快开启基本实现现代化新征程的重要一年。做好今年的工作意义重大。尽管世界经济复苏的不稳定性、不确定性上升，国内经济保持平稳较快增长的压力加大，经济发展面临的形势将更为严峻，带来的挑战和考验不可低估，但就连云港而言，总体上仍然是机遇大于挑战。我们的机遇是，我国经济发展长期向好的基本态势没有改变，国家已经释放出对宏观调控适时适度预调微调的积极信号，宏观政策环境将进一步趋好；我市拥有的四个国家战略聚焦叠加的效应不断释放，国家和省委省政府对我市的支持力度将进一步加大。特别是市第十一次党代会为我们加快沿海崛起、实现新的跨越指明了方向和路径，全市上下抓转型、促发展的干劲更加高昂，加快崛起振兴的基础更加坚实，经济社会持续快速发展的势头更加强劲。只要我们始终保持时不我待的机遇意识，克难奋进的顽强斗志，勇争一流的拼搏精神，奋起争先，昂扬前进，就一定能够实现新跨越，夺取新胜利!

根据中央、省经济工作会议精神和市委十一届二次全会部署，今年政府工作总的要求是：坚持以邓小平理论和三个代表重要思想为指导，深入贯彻落实科学发展观，认真实施省委八项工程，以全面建成小康社会、加快推进基本现代化为目标导向，围绕推进沿海开发、加快转型升级年度工作主题，坚持稳中求进、能快则快、又好又快的原则，加快港口建设，做大做强产业，统筹城乡发展，加强三农工作，深化改革开放，推动经济平稳快速增长，不断增进民生幸福，全面开创经济社会又好又快发展新局面。

主要预期目标是：地区生产总值增长13%左右，三次产业结构调整为12.4　:　47.8　:　39.8，财政一般预算收入增长20%，固定资产投资增长23%，城镇居民人均可支配收入和农民人均纯收入均增长13%左右，城镇登记失业率控制在3%以内，居民消费价格水平不高于省控标准，节能减排完成省下达任务。

新的一年，市政府将坚持把增进民生幸福放在更加突出的位置，把70%以上的公共财力投入到民生领域，扎实推进城市快速公交、饮用水保障、棚户区改造、保障房建设、校舍安全、农贸市场改造、专类公园、卫生信息平台、全民健身、社会福利中心等十大民生工程，让改革发展的成果真正落实到富民、惠民、安民上。

围绕上述目标和要求，着力做好八个方面工作：

一、奋力突破沿海开发，加快构筑经济发展新优势

坚持以基础设施为支撑、以示范区建设为带动、以政策项目为推动，着力突破沿海开发，加快把政策支持优势转化为现实发展优势。

加速一体两翼港口建设。启动30万吨级航道二期工程前期工作，建成连云港区25万吨级航道和徐圩、赣榆港区5万吨级航道，建成灌河港区口门2万吨级航道主体工程。连云港区旗台防波堤投入使用，大堤作业区2个10万吨级集装箱泊位开工建设。加快徐圩港区防波堤、2个10万吨级散货码头建设。基本建成赣榆港区4个5万吨级码头并开港运营。建成连云港区中云内河港29个泊位。进一步完善集疏运体系，全面加快北疏港道路、矿石输送机隧道工程建设，基本建成30万吨级矿石码头配套铁路专用线。加大交通、电力等基础设施投入，加快临海高等级公路及310等国省道建设改造，完成盐河航道整治主体工程。基本完成连盐铁路征地拆迁，争取全线开工建设。加快500千伏临海、220千伏洋桥、110千伏中云等输变电工程建设，加大农村电网改造力度，保障生产生活用电需求。

加快推进国家东中西区域合作示范区建设。编制建设行动方案，突出抓好先导区建设。强势推进徐圩新区重大产业、功能平台、路网及配套设施建设。启动建设大陆桥合作论坛永久会馆、产品展览展示中心。全线开工建设徐新公路，贯通港前大道路基。开工建设热电中心。建成徐圩送水工程、自来水厂和污水处理厂一期工程。编制现代物流、产业合作、商务服务、科技人才和生态功能区专项规划，统筹推进五大功能区建设。进一步加强中西部需求分析，强化要素资源对接，积极探索区域合作共建和利益共享机制。推动国家层面建立部省联席会议制度。推进陇海兰新铁路建立运输协调服务机制，推动陆桥物流、沿线地区口岸查验单位一体化发展。

加大政策和项目落实推进力度。围绕重大产业、基础设施、政策支持、园区载体和组织保障等五大类50个重大项目和重要事项，积极对接争取。深入推进大型石化、钢铁基地、中日（连云港）生态科技产业园、沿海发展银行、国家开发银行连云港分行等一批重大项目和事项，尽快取得实质性突破。大力推动徐连客运专线、连淮扬镇铁路前期工作，积极申报综合保税区，争取新机场立项、开通日韩航线和两翼港区开放，做好中日韩自由贸易区先导区争取工作。

二、实施转型升级工程，加快构建现代产业体系

坚持增量带动与存量提升并重，壮大新兴产业与发展临港产业并举，促进产业布局合理化、发展集聚化、结构高端化。

推动工业集中集聚发展。实施双千双百工程，重点打造新医药、新材料、新能源、高端装备、石化、冶金等千亿支柱产业，培育市开发区、徐圩新区、赣榆临港产业区、灌云临港产业区、灌南临港产业区、东海经济开发区等千亿园区，引导县区培育百亿特色产业、百亿龙头企业，年销售收入过百亿企业达4家。突出高端引领，加快发展战略性新兴产业，进一步提升三新产业、高端装备制造业市场规模和技术领先优势，培育壮大新信息技术、节能环保和海洋产业，注重发挥海洋资源丰富的优势，大力发展海洋生物医药等海洋新兴产业。继续实施高新技术产业倍增计划，产值达到1300亿元。加快推进基础性、基地型临港工业集聚，抓好环球铜业、新海石化三期等重大工业项目建设，石化、冶金产业产值均增长30%以上。开展千企升级行动，引导中小企业走专精特新发展路子，重点实施100项技术改造项目，加快推动制造业向高端发展。完成工业投资920亿元，工业总产值达3500亿元，规模以上工业增加值增长17%。

加快服务业扩张升级。重点抓好核心商圈、区域性专业市场、消费市场、特色街区建设，实施49个服务业项目，打造10个现代服务业集聚区，促进服务业扩量提速、比重提高、竞争力提升。服务业增加值增长14%。以培育千亿物流产业为重点，大力发展生产性服务业。启动航运中心集聚区建设，增辟6条集装箱航线，新增2条五定班列、1个内陆无水港，完善口岸服务平台和铁水联运等公共信息网络，完成港口吞吐量1.8亿吨，集装箱运量560万标箱。民航客运量增长10%以上。积极构建临港物流集聚区，加快金港湾物流园等项目建设，培育煤炭、矿砂、粮食等大宗物资中转配送基地，做大做强木材贸易加工等特色物流园区，完善两翼港区物流体系。推进区域性商务中心建设，提升中央核心区商务功能。以提高生活品质为目标，积极发展生活性服务业。增强文化自觉和文化自信，大力发展文化产业。加快市广电、报业、演艺集团等组建工作，扎实推进西游记文化产业园、广播影视城、联创酷歌动漫产业园建设，打造水晶文化、徐福文化创意产业园。深度开发山海文化旅游资源，加强城市旅游策划和形象推广，推动花果山5A级景区创建，提升景区配套设施功能，游客接待量、旅游总收入分别增长15%和20%。突出打造城区商贸集聚区，建设苏宁广场等一批重点项目，进一步优化城区商业布局。加快电子商务、连锁经营、物流配送等现代服务业态发展，强化农村商品流通网络建设，农家店行政村覆盖率达到95%。鼓励扩大文化、娱乐、健身等多样化服务消费，切实改善城乡消费环境。

引导农业高效规模发展。实施农业现代化工程，提升科技兴农水平，开展特色产业基地、粮食高产增效、一村一品、规模养殖、农业园区五大创建活动，积极培育国家级现代农业园区。加大农业综合开发投入，加强以农田水利为重点的农业基础设施建设，建立万亩高产增效示范片50个。新增设施农业10万亩，发展优质林果茶基地4.5万亩，畜禽规模化养殖比重提高5个百分点。建设深水网箱养殖示范基地，打造海州湾百亿现代综合渔业园区。推进食用菌、鲜切花、设施蔬菜等产业做强做优，争取四县各培育1个产值过10亿元的农业特色产业，每个乡镇争创1家市级农业园区。引导农产品加工企业集群集聚，新办规模以上龙头企业100家，农产品出口增长15%。发展适度规模经营，农户参加专业合作经济组织比重达45%。完善农产品市场体系和信息服务体系，农产品现代流通业态销售率达到66%。健全质量安全监管体系，提高技术装备水平，创建省级名牌农产品6个、国家地理标志农产品3个。

三、强化功能提升，加快建设现代化国际海港中心城市

优化城市空间布局，增强综合服务功能，把控建设时序，坚持建管并重，着力提升城市品质内涵。

提高城市规划水平。注重规划的连续性、系统性和先进性，编制完善城市控规、修规和城市设计，细化城市组团及功能片区的建筑风格、色彩、结构形态、公共设施配套要求。优化深化连云新城商务中心区、科教创业园区、徐圩新区、滨河组团、盐河组团、龙河核心商业区等区域的城市设计。加强规划管理，发挥导向作用，建立城市规划分层管理机制，合理配置土地、岸线等要素资源，提升城市资源整合和利用效率。

推进城市组团开发。集中优势资源，突破重点片区，推进各个组团快速形成规模。东部城区着重彰显海滨特色，加快集聚商务、金融、航运、休闲度假等功能，为大港口、大产业发展提供配套。新海城区着重提升功能品质，增强面向居民生活的商业、文化、科教等宜居功能。花果山大道沿线重点拓展科研教育、文化旅游、商务办公等功能，为高新技术产业、现代服务业发展提供支撑。创新城市建设管理体制，明确市、区共建城市的分工定位。市级层面，东部城区重点突破连云新城，抓好5平方公里的新城商务区建设，实施7个陆域形成项目、36个市政工程和2个公用建筑项目；新海城区统筹规划建设科教创业园区、行政中心片区、凤凰片区，实施34项道路桥梁工程，开工建设卡迪亚国际广场等重点项目，集中精力打造一片新城；徐圩新区适应产业发展配套需要，着力打造集商务服务、科技研发、生活居住、旅游文化四大功能为一体的高端配套服务区。区级层面，重点推进滨河组团、盐河组团、连云国际商务中心组团建设。中心城市完成城建投入283亿元，建成区面积扩大到140平方公里。城市化率达55%。

增强城市综合功能。围绕提升中心城区集聚辐射能力，加快完善城市基础设施和公共配套，实施路网改造、刘湾垃圾填埋场一期工程等81个重点城建项目。推进城市快速公交体系建设，建成快速公交1号线，促进新海城区与东部城区融合发展。实施郁州路下穿等城市主次干道和桥梁工程100项，畅通城市道路微循环。开工建设市第三水厂扩建工程，新建改建城区供水管网30公里，新增城市污水管网50公里，城市污水处理率达83%。提高配套设施便民程度，整治老旧道路、背街小巷30条，新建街头游园20个，小区物业管理覆盖率达83%。提高城市防洪排涝能力，推进供电、供热、管道燃气、通讯、民防等设施建设。实施市档案馆新馆、公共卫生中心等文教卫配套项目15项，建成康达学院和全民健身中心。

提升城市特色和品质。充分挖掘城市内涵，做好海州古城、南城古镇、连云古镇等综合保护利用，打造特色鲜明、功能完善的历史文化街区，延伸城市文脉，留住城市记忆。彰显生态宜居城市魅力，加快推进山海景观轴、云湖公园、孔望山公园二期、临洪河口湿地等一批生态公园建设，新增城市绿地300万平方米，城市绿化覆盖率达41%。深入推进四城同创，抓好重点地段、重点区域、重点道路的整治出新，加快推进黄海度假村、连盐铁路周边等改造项目，加大禁违拆违力度，完成旧城改造面积50万平方米。大力开展市容环境综合整治，提高环卫保洁水平，加大对道路抛洒、乱涂乱贴乱放等重点问题的整治力度，实现全市生活垃圾无害化处理全覆盖。规范配置停车场、街巷路灯、交通标识，下大力气改善主城区交通拥堵、违章行驶、占道经营等状况。完善数字化城市管理体系，推动城市管理重心下移，建立健全城市管理快速反应和长效管理机制，切实提高城市管理效能。

四、坚持改革开放，加快推进体制机制创新

用开放创新的办法，汇聚优质要素资源，破解发展难题，为科学发展注入活力、拓展空间、增强后劲。

推进创新型城市建设。深入实施创新驱动战略，建立创新型城市建设目标责任考核体系，进一步提高全社会研发支出占地区生产总值的比重，科技进步贡献率达到45%。推进区域重大创新平台建设，建成中科院能源动力研究中心、省海洋资源开发研究院，建设国家口岸药品检验所。支持省内外高校、科研院所在连兴办研究院，建设5个专业化产学研合作平台，支持七一六研究所与地方互动合作。提升企业创新能力，新培育高新技术企业40家，发展民营科技企业1600家，新增工程技术研究中心、院士工作站等研发机构30家。大力推进质量强市，实施技术标准战略，争创自主品牌，专利申请量、授权量分别达3000件和1600件。积极实施人才集聚工程，创新人才培养引进模式，加快培养企业急需、社会紧缺的技能型人才，引进高层次创业创新人才100名、领军人才10名，提供人才安置房100套。推进省级高新区升格为国家级高新区，力争东海县进入省第二批创新型试点城市。

大力发展开放型经济。加大宣传推介力度，营造浓厚招商氛围，全面打响招商引资攻坚战，境外招商主攻日韩港台、巩固拓展欧美，重点强化专题招商，充分利用承办第十二届泛黄海中日韩经济技术交流会议等大型活动，提高招商活动针对性和组织化程度。强化产业链招商，围绕优势产业、临港基础产业、战略性新兴产业和现代服务业，抓好72个重大招商引资项目，争取早落户早投产。优化外企服务，促进增资扩股。直接利用外资8亿美元，内联客方到位资金560亿元。培育壮大机电产品和科技兴贸出口基地，外贸出口总额增长25%。支持企业开展境外投资，大力发展国际服务贸易。推进中小企业园建设。编制开发园区产业发展及空间布局规划，建立健全园区考核评价体系，明确主导产业，引导关联企业集中布局，提高投资强度。制定园区建设标准，完善园区服务配套功能，搭建物流、科技、人才等公共服务平台，合理布点工业邻里中心。争取一批开发园区扩容升格，做好中德中小企业园、盐化科技产业园等特色园区建设工作。

推进重点领域改革创新。深化行政管理体制改革，开展扩权强镇试点，柘汪镇、安峰镇争创国家综合改革试点镇。推进医药卫生体制改革，巩固完善基本药物制度，探索公立医院改革路径。稳步推进水电气价格改革，推行居民生活用水、用电阶梯式计价。争创省级金融创新示范区，引进和组建金融机构10家以上，实现四县村镇银行和农商行全覆盖。推进区域集优直接债务融资，争取发行企业债券2支。推进优质企业上市融资，新增上市公司2家。规范融资市场秩序，强化融资性担保机构监管，新增中小企业贷款100亿元。加强社会信用体系建设，建立完善公共信用信息系统。开展农村产权改革，每个县区选择1-2个乡镇进行试点，落实土地承包经营权、集体建设用地使用权、农民自有住房抵押贷款等配套政策。大力推动全民创业，民营经济增加值增长14%。

五、促进绿色增长，加快打造生态宜居新家园

深入实施生态文明建设工程，统筹资源开发与环境保护，进一步彰显连云港的山水之美、自然之美、绿色之美。

大力推进节能减排。建立节能减排联席会议制度，严格执行投资项目节能减排评估审查、区域限批等制度，把住源头，严控增量，加快形成以低碳为特征的产业体系。开展冶金、电力等重点耗能企业节能行动，鼓励引导企业采用先进技术和设备改造升级。实施重点节能技术改造项目40个、重点减排项目30项，创建节能和循环经济示范工程10项。加大节能减排基础设施投入，加强日常监管，健全监测预警、统计和考核体系，促进各项措施落到实处。

加强环境综合整治。实施清水蓝天工程，提高人民群众对生态环境的满意度。落实最严格的饮用水源保护措施，按照双源双线双湖城市饮用水保障体系规划，实施蔷薇河沿线控污、治污等工程，饮用水水质达到国家安全标准。继续实施农村饮用水安全工程，新增9万农村饮水安全人口。全面实施农村河道疏浚，加强农村生产生活污水和农业面源污染治理，新建20个乡镇污水处理厂和垃圾中转站，完成20%的村庄整治任务。加大大气污染治理力度，抓好秸秆禁烧、废气污染等重点问题整治，增强对大气中细颗粒物的监测预警能力，控制灰霾等空气污染，市区空气质量优良率达85%以上。加强化工行业、重点园区等关键领域环境问题管控，保障环境安全。

强化生态建设。围绕创建生态市目标，切实加强云台山自然保护区建设，抓好东西连岛、秦山岛的生态保护性开发，扎实推进沿海滩涂、破损山体、工矿废弃地综合治理和植被恢复，加大森林保护和城乡绿化力度，新增造林面积12万亩，森林覆盖率达到24.5%。全面启动海州湾国家级海洋公园规划建设，加强海洋环境保护和生态修复，海洋牧场面积达到15万亩。严格土地管理制度，落实耕地保护责任制，健全依法用地长效机制，提高土地节约集约利用水平。创建5家国家级生态乡镇和10家省级生态乡镇。

六、实施民生幸福工程，加快全面小康社会建设

坚持以人为本、民生优先，以全面建成小康社会为总目标，实施一个计划、构建六大体系，让人民群众生活得到新改善、实现新提升。

加快实施收入倍增计划。把促进就业作为经济社会发展的优先目标，加大财税、信贷等政策对创业就业的支持，新增城镇就业8万人、农村劳动力转移3万人以上，确保登记困难家庭高校毕业生100%就业，实现城镇零就业家庭动态为零。加强职业技能培训，完成城乡职业培训25万人次、职业技能鉴定4万人，对农民创业培训辅导全免费。实施新一轮脱贫奔小康工程，落实最低工资标准，继续提高企业退休人员基本养老金，扩大中等收入阶层比重。城镇居民人均可支配收入、农民人均纯收入分别达到20500元和9300元。

提高民生保障水平。加快构建终身教育、就业服务、社会保障、基本医疗卫生、住房保障、养老服务六大体系，推动民生持续改善。实施学前教育五年行动计划，推进义务教育优质均衡发展，初中毕业生升学率稳定在96%以上，基本实现教育现代化。支持淮海工学院做好硕士点建设，依托广播电视大学建设社区大学。推动职业教育提升水平、创出特色，连云港中专投入使用。健全就业服务体系，推进公共就业服务平台建设，创建9个省级充分就业示范社区。进一步完善各项社会保障制度，扩大社会保险覆盖面，五险参保人数平均新增1.6万人以上，适时提高失业保险、新农保和城镇居民社会养老保险基础养老金标准，被征地农民社会保障覆盖率90%以上，城乡居民医疗、养老保险参保率分别达95%和98%以上。完善城乡卫生服务网络，建设全科医生队伍，创建三星级以上基层医疗卫生机构35家，建成市县一体化区域卫生信息平台，实现全市就医一卡通。加强住房保障，扩大住房公积金覆盖面，开工建设各类保障性住房10500套，发放廉租住房租赁补贴1950户，确保分配管理公开公平公正。推进锦屏磷矿、金桥盐化棚户区改造，分别建成住宅1600套和2200套。加强社会养老体系建设，保障老年人权益，大力发展养老机构，新增养老床位500张以上，农村五保对象集中供养率70%以上。

推进以县为单位的全面小康社会建设。加快发展壮大县域经济，推动各县在全省进位争先。提升城乡基础设施、基本公共服务一体化水平，实施科教文卫等十大下乡工程，完成农业三资投入100亿元以上。提高四县县城规划建设水平和集聚带动能力，确保新区开发见成效、旧城改造出形象。实施中心镇提升工程，稳妥推进镇村区划调整，重点打造一批工业强镇、沿海重镇、现代农业大镇和乡村旅游名镇。加快建设美好城乡，按照布局合理、设施配套、环境宜居、特色鲜明的要求，每个县重点建设2个重点中心镇和一批农村新型社区。加强全面小康进程监测和评价考核工作，确保各项小康指标按序时进度推进并不断巩固提升，赣榆、东海达到全面小康标准。

七、推进社会管理创新，加快社会事业发展

主动适应经济社会转型升级的新要求，不断提高社会事业建设管理水平，为人民群众提供更多更好的公共服务。

推动文化事业繁荣发展。大力实施文化建设工程，积极发展群众喜闻乐见的文化、体育、娱乐新业态，加快构建覆盖城乡的公共文化服务体系。深入实施文化惠民工程，巩固乡镇文化站、社区文化中心、农家书屋建设成果，广泛开展和谐文化进万家活动。完善公共体育服务体系，构建城市十分钟健身圈。打造文化精品，深入挖掘山海、淮盐、西游和民俗等地方文化内涵，策划、包装一批特色文化项目，扶持一批重点文化品牌、文化名人，增强地域文化影响力。

强化社会管理和公共服务。深入实施社会管理创新工程，完善社会管理工作格局。深化平安创建活动，加强社会稳定风险评估，健全完善矛盾纠纷大调解机制，落实信访责任制，着力解决影响和谐稳定的源头性、根本性、基础性问题，形成具有连云港特色的社会管理体系。加强和改进人口服务管理，推进世代服务提质工程全覆盖。强化食品药品监管，深入开展食品安全专项整治，创建国家和省级药品安全示范区。落实安全生产责任制，实现隐患治理制度化，加强中小学校舍、校车投入和安全监管，坚决遏制重特大事故发生。加强应急管理体系建设，提升突发事件应急处置能力。稳控物价水平，加大农超对接力度，新增10家菜篮子生产基地、20家平价商店，完成10个农贸市场改造，着力解决农民卖菜难和市民买菜贵的问题。

加强精神文明和民主法制建设。大力弘扬社会主义核心价值观，实施公民思想道德建设示范引导工程。深化未成年人思想道德建设，完善学校、社会、家庭三位一体的教育网络。深入开展创业创新创优、争先领先率先主题教育活动。积极倡导志愿服务精神，不断提高社会文明程度。坚持依法治市，深入开展六五普法工作，加强基层民主建设，提升法治连云港建设水平。扎实做好国防动员、民兵预备役和优抚安置工作，提高双拥工作水平。继续发展妇女儿童事业，健全残疾人服务体系，推动防震减灾、民族宗教、地方志、气象、老区开发、关心下一代、红十字、慈善、光彩事业再创新成绩。

八、牢固树立宗旨意识，切实加强政府自身建设

新的形势和任务，对政府工作提出了新的、更高的要求。我们将更加重视政府自身建设，全面增强狠抓落实、服务发展的能力，确保各项目标任务圆满完成。

进一步增强制度约束力，提高组织化程度，又快又好地推进工作落实。认真落实重大事项集体决策、专家咨询、社会公示和听证制度，对规范性文件实施情况加强跟踪检查和评估反馈。严格执行规划管理、土地储备与出让、重大资金使用集体会办等制度和机制，提升规范管理和整合利用水平。完善目标管理，明确工作标准，量化目标任务，细化责任分解，加强绩效考核。加大行政问责力度，严肃查处不作为、乱作为等行为，切实提高行政效能。

进一步改进机关作风，坚持勤政为民，优化政务服务。深化行政审批制度改革，强化公共服务职能，实行限时服务、承诺服务、跟踪服务，积极主动地为基层和企业解难题、办实事，切实优化投资发展软环境。健全四级便民服务网络，充分发挥12345服务热线作用，及时处置群众反映的问题。加强作风建设，大力改进会风、文风，用更多的时间调查研究、真抓实干，努力提升机关服务水平。

进一步推进依法行政，加强廉政建设，始终保持清廉务实的良好形象。健全政务公开、办事公开、新闻发布等制度，完善权力阳光运行机制。自觉接受人大法律监督、工作监督和政协民主监督，按照法定权限和程序行使权力、履行职责。严格执行廉洁从政责任制，健全重大事项申报制度，完善电子监察系统，强化对重点领域、重点资金、重点项目的审计监督。严控公用经费和一般性支出，坚决制止铺张浪费。

各位代表，我们正处于加快沿海崛起，实现新的跨越的关键时期，困难和挑战考验着我们，责任和使命激励着我们。让我们在中共连云港市委的坚强领导下，开拓创新，扎实工作，为全面建成更高水平小康社会、开启基本实现现代化新征程而努力奋斗！
